# Selective flow-induced vesicle rupture to sort by membrane mechanical properties

**DOI:** 10.1038/srep13163

**Published:** 2015-08-25

**Authors:** Angelo Pommella, Nicholas J. Brooks, John M. Seddon, Valeria Garbin

**Affiliations:** 1 Department of Chemical Engineering, Imperial College London, London SW7 2AZ, United Kingdom; 2 Department of Chemistry, Imperial College London, London SW7 2AZ, United Kingdom

## Abstract

Vesicle and cell rupture caused by large viscous stresses in ultrasonication is central to biomedical and bioprocessing applications. The flow-induced opening of lipid membranes can be exploited to deliver drugs into cells, or to recover products from cells, provided that it can be obtained in a controlled fashion. Here we demonstrate that differences in lipid membrane and vesicle properties can enable selective flow-induced vesicle break-up. We obtained vesicle populations with different membrane properties by using different lipids (SOPC, DOPC, or POPC) and lipid:cholesterol mixtures (SOPC:chol and DOPC:chol). We subjected vesicles to large deformations in the acoustic microstreaming flow generated by ultrasound-driven microbubbles. By simultaneously deforming vesicles with different properties in the same flow, we determined the conditions in which rupture is selective with respect to the membrane stretching elasticity. We also investigated the effect of vesicle radius and excess area on the threshold for rupture, and identified conditions for robust selectivity based solely on the mechanical properties of the membrane. Our work should enable new sorting mechanisms based on the difference in membrane composition and mechanical properties between different vesicles, capsules, or cells.

Lipid vesicles are useful models for cell deformation, for instance to mimic the flow of red blood cells^1^ or white blood cells^2^ inside capillaries. The dynamics of vesicles upon small deformation in a flow has been investigated extensively^3^,^4^,^5^,^6^,^7^,^8^, and different regimes have been revealed, such as tank-treading^4^, trembling^3^, and tumbling^5^, depending on the vesicle properties and the flow parameters. Flow-induced deformation of vesicles typically occurs at constant volume and area, as the lipid membrane enclosing a vesicle can be considered to be inextensible in this regime^9^. Upon large deformation, the membrane is stretched^10^, and the vesicle breaks when a critical rupture tension is reached^11^,^12^. This behavior has been studied in micropipette aspiration experiments, but it has rarely been studied for the case of flow-induced deformation.

Flow-induced opening and rupture of cell membranes is of central importance in biomedical applications^13^,^14^, where pore formation can enhance drug uptake, and in bioprocessing^15^,^16^, where cell disruption is necessary to recover materials produced by the cells. In these applications, large viscous stresses are generated due to cavitation and bubble dynamics during ultrasonication. So far, only a few studies have reported direct, time-resolved observations of vesicle and cell rupture in the complex flows generated by ultrasound-driven bubbles^17^,^18^,^19^. These studies have revealed different rupture mechanisms depending on the flow regime, namely acoustic microstreaming^17^,^18^ or bubble collapse with high-speed liquid jet formation^19^. To exploit cell and vesicle rupture in a controlled fashion in applications, a better understanding of these mechanisms is required. While it is well established that the interplay between viscosity ratio, membrane viscosity^3^, bending modulus^20^, and vesicle excess area^21^ governs the behavior upon small deformation, the role of the vesicle and membrane properties in vesicle rupture remains largely unexplored. Intriguingly, demonstrations of capsule and cell sorting based solely on the membrane elastic properties^22^,^23^ suggest that it should be possible to identify conditions in which vesicle rupture is selective with respect to the mechanical properties of the membrane. These properties depend on the composition of the lipid bilayer^10^,^11^,^12^,^24^,^25^, on temperature^26^ and pressure^27^. Since the membrane composition varies significantly between different cell types and between organelles within a cell^28^, and can be altered by diseases^29^, mechanical selectivity of vesicle rupture would open up the potential for new sorting methods.

Here we demonstrate experimentally the mechanical selectivity of flow-induced vesicle rupture with respect to the membrane stretching elasticity. We tuned the mechanical properties of giant unilamellar vesicles by tuning the lipid composition of the membrane using different lipids (SOPC, DOPC, POPC) and lipid:cholesterol mixtures (SOPC:chol, DOPC:chol). We subjected the vesicles to strong deformations, leading to rupture, in the acoustic microstreaming flow generated by microbubbles driven by ultrasound. In addition to mechanical selectivity, we observe a size threshold for rupture within the same vesicle population. We interpret our observations in terms of a capillary number based on the stretching elasticity^18^. The experimental data for the maximum viscous stress at rupture show that the vesicle excess area can influence the rupture conditions. We characterized the excess area distributions of the five vesicle populations, and identified conditions in which the variability in excess area does not affect mechanical selectivity. Together, these observations suggest that it is possible to identify conditions for robust mechanical selectivity of flow-induced break-up of soft objects—vesicles, capsules, or cells—with different membrane properties.

## Results and Discussion

### Vesicle deformation in acoustic microstreaming flow

**Figure 1:**
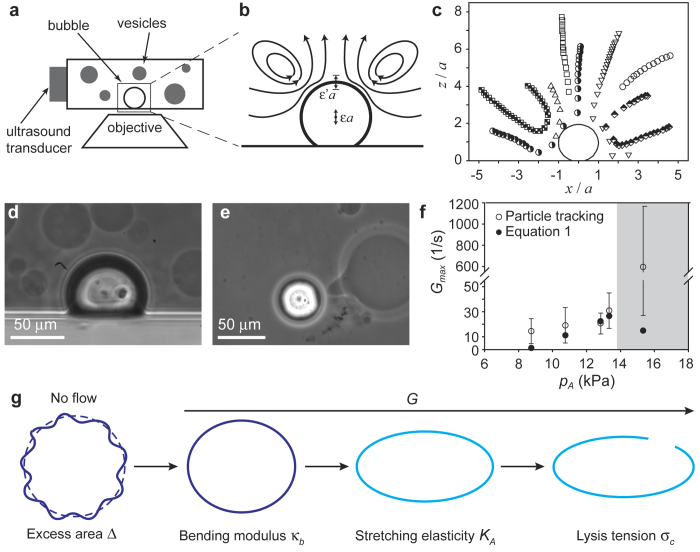
Vesicle deformation in acoustic microstreaming flow. (**a**) Schematic of the experimental setup (not to scale). (**b**) Sketch of the streamlines of the acoustic microstreaming flow, showing the stagnation points at the center of the closed streamlines near the bubble. (**c**) Experimental streamlines of the acoustic microstreaming flow determined by particle tracking. (**d**) Side view of the vesicle suspension around the adhered microbubble. (**e**) Top view showing deformation of a vesicle in the microstreaming flow, particularly on the side that is closest to the bubble. (**f**) Comparison of the strain rate, *G*_max_, determined experimentally by particle tracking (open symbols), with the estimates obtained from Equation (1) (solid symbols). (**g**) Regimes of vesicle deformation for increasing strain rate *G*.

We observed the deformation and rupture of vesicles in the acoustic microstreaming flow generated by an ultrasound-driven microbubble oscillating near a solid wall (see Fig. 1). The bubble adheres to the wall of a quartz cuvette as shown in Fig. 1a and is driven into radial oscillations by an applied ultrasound wave with acoustic pressure amplitude *p*_A_ (see *Methods* for details). The radial oscillations of the bubble near a rigid wall, with amplitude *ε*′*a*, where *a* ≈ 10–100 *μ*m is the bubble radius, result in translational oscillations of the center of mass, with amplitude *εa*^30^. These radial and translational oscillations produce a non-linear secondary flow with closed streamlines^30^ as shown in Fig. 1b. Figure 1c shows experimental streamlines of the microstreaming flow generated by a bubble undergoing small-amplitude, spherical oscillations. As vesicles are transported along the streamlines they collect in the vicinity of the bubble (Fig. 1d). Vesicles that become trapped in the stagnation point of the microstreaming flow remain close to the oscillating microbubbles and experience significant deformation, particularly on the side that is closest to the bubble (Fig. 1e). The microstreaming flow presents components of both shear and elongation with complex spatial dependence^18^, and can be characterized by the total strain rate *G*. For spherical oscillations, the viscous stresses in the microstreaming flow can be estimated from the bubble dynamics parameters. For a sphere executing radial and translational oscillations with relative amplitudes *ε*′ and *ε* respectively, the maximum strain rate is given by


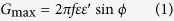


where *f* is the frequency of oscillations and *ϕ* is the phase shift between the radial and translational oscillations^31^. We measured the maximum strain rate from experimental streamlines for spherical oscillations, and compared the experimental values with the results of Equation (1) (see Fig. 1f). The simplified model correctly captures the order of magnitude of the maximum strain rate for oscillation of small amplitude (*ε*, *ε*′ ≪ 1). The equation is not applicable when the oscillations become non-linear, that is, for sufficiently large acoustic pressure amplitude (*p*_A_ ≈ 14 kPa in the example of Fig. 1f, see shaded area). We can therefore estimate the maximum strain rate in the linear regime by measuring the bubble dynamics parameters, *ε* and *ε*′, and using Equation (1). The error introduced by using this approximation will be taken to be the typical relative deviation of Equation (1) from the particle-tracking results, as determined in Fig. 1f.

The magnitude of the strain rate, *G*, determines the regime of vesicle deformation (Fig. 1g). Vesicles at rest are characterized by an excess area Δ, defined as 
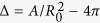
, with *A* the vesicle external area, and *R*_0_ the radius of a sphere with the same volume. As the vesicle is subjected to an external flow, the undulations of the membrane are flattened. This deformation occurs at constant external area and is governed by the membrane bending modulus *κ*_b_. For larger strain rate, such that the membrane is stretched, the membrane stretching elasticity *K*_*A*_ governs the deformation, until the critical tension, *σ*_c_, is reached and the vesicle breaks. The deformation under flow of a vesicle that is undergoing stretching of the membrane can be described by a capillary number based on the stretching elasticity,


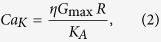


with *η* the bulk viscosity and *R* the vesicle radius. The capillary number describes the balance between the viscous stresses deforming the membrane, and the elastic stresses resisting deformation. The model for vesicle deformation introduced in Ref. 18, which takes into account stretching of the membrane, predicts a threshold value of the capillary number for vesicle rupture:


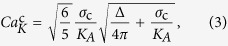


which depends on the membrane mechanical properties and the vesicle excess area. For a given vesicle radius *R*, and given flow conditions *η* and *G*_max_, vesicles with larger stretching elasticity, *K*_*A*_, exhibit a smaller capillary number, 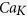, i.e., they deform to a smaller extent. For a vesicle to break, it has to deform to the point that the maximum area dilation, *α* = *σ*_c_/*K*_*A*_, is exceeded (Fig. 1g). In particular, the deformation has to be sufficiently large that the undulations of the membrane due to the excess area, Δ, are “ironed out”, and the membrane is stretched so that the area dilation exceeds the threshold *α*. The combined effect of excess area and maximum area dilation is described by the critical capillary number 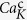. The break-up criterion can therefore be expressed as 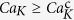
18.

We made giant unilamellar vesicles with different membrane mechanical properties by tuning the lipid composition. We made vesicles with single-component lipid membranes (SOPC, DOPC, or POPC) and with mixtures of lipids and cholesterol (SOPC:chol, or DOPC:chol). The compositions used give homogeneous liquid phases at room temperature. Table 1 compares the literature values of bending modulus *κ*_b_, stretching elasticity *K*_*A*_, and the maximum area dilation *α* = *σ*_c_/*K*_*A*_, for the five vesicle compositions used^10^,^11^,^12^,^24^,^25^,^26^. To evaluate the critical capillary numbers 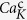 for the five vesicle populations, we used the literature values for the mechanical properties, and measured the excess area Δ in capillary flow experiments (see *Methods*). The experimentally determined average value of the excess area for each population is reported in Table 1. The resulting values of the critical capillary numbers, calculated using Equation (3), are also reported in Table 1.

### Selective rupture of vesicles with single-component lipid membranes

**Figure 2:**
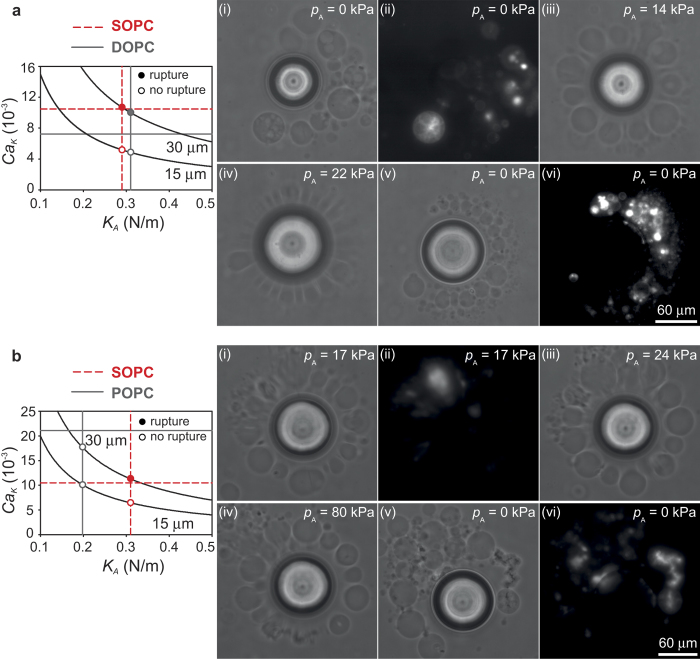
Selectivity of flow-induced vesicle break-up for single-component membranes. (**a**) SOPC (fluorescent) against DOPC. Frames (i–vi) show no selectivity, as vesicles from both populations break in the microstreaming flow. The graph compares the critical capillary numbers (horizontal lines) and stretching elasticities (vertical lines) for SOPC (dashed lines) and DOPC (solid lines). For *R* ≈ 30 *μ*m 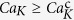 for both SOPC and DOPC. (**b**) SOPC (fluorescent) against POPC. Break-up is selective, as all SOPC vesicles with *R* > 15 *μ*m break, while most POPC vesicles remain intact in frames (i–vi). The graph shows that 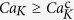 only for SOPC vesicles (dashed lines) but not for POPC vesicles (solid lines). All images are in top view.

To test the selectivity of vesicle break-up with respect to the mechanical properties of the membrane, we simultaneously deformed vesicles with different lipid composition in the same acoustic microstreaming flow. Figure 2a shows an experiment where pure SOPC vesicles are compared with pure DOPC vesicles. The SOPC vesicles are fluorescently labelled (see *Methods*), while the DOPC vesicles are not fluorescent. Frames (i-ii) in Fig. 2a show the configuration of vesicles around the bubble before the microstreaming flow is activated (*p*_A_ = 0 kPa). The phase contrast image in frame (i) shows several vesicles ranging in radius from a few microns up to 25 *μ*m. The fluoresence image in frame (ii) shows only the SOPC vesicles. When the microstreaming flow is activated, with an acoustic pressure *p*_A_ = 14 kPa, the vesicles are slightly deformed as shown in frame (iii). Increasing the acoustic pressure to *p*_A_ = 22 kPa causes significant deformation and rupture of the vesicles as shown in frame (iv). Frames (v-vi) show the configuration after the microstreaming flow has stopped (*p*_A_ = 0 kPa). SOPC and DOPC vesicles larger than 15*μ*m have ruptured, and only small SOPC and DOPC vesicles are present. Small vesicles can either be the product of the break-up of larger vesicles, or they were initially present and have not ruptured. These observations are consistent with the magnitudes of the capillary numbers, 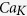, and the critical capillary numbers, 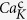, for SOPC and DOPC membranes. The graph in Fig. 2a reports the capillary number, *Ca*_*K*_ = *ηG_max_R*/*K*_*A*_, as a function of *K*_*A*_, for two different vesicle radii, *R* = 15*μ*m and *R* = 30*μ*m. The other parameters are representative of typical conditions in the experiments, i.e., a characteristic stress *τ*_max_ = *ηG*_max _≈ 100 Pa. For the experiments of Fig. 2, it is not possible to obtain *τ*_max_ from Eq. (1), because the bubble oscillations are non-linear. Estimates of *τ*_max_ that motivate the assumption made here are provided later in the paper. The horizontal lines represent the values of the critical capillary number, 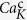, for SOPC (dashed line) and DOPC (solid line). The critical capillary number depends only on the properties of the membrane, and not on the vesicle size or the flow conditions. For vesicles with *R* = 30 *μ*m, the capillary numbers for both SOPC and DOPC vesicles exceed the respective thresholds for break-up (solid circles). For radii *R* = 15*μ*m and smaller, both SOPC and DOPC vesicles exhibit capillary numbers below the respective thresholds for break-up (open circles). We observed approximately 10 SOPC vesicles and 20 DOPC vesicles, and found probabilities of break-up of vesicles larger than 15 *μ*m in the same flow conditions of 70% and 65%, respectively. Because SOPC and DOPC vesicles present similar values of stretching elasticity, *K*_*A*_, and of the critical capillary number (see Table 1), selective break-up of only one population is not possible.

In contrast, the properties of SOPC and POPC vesicles are significantly different (see Table 1). The stretching elasticity of POPC vesicles is smaller than that of SOPC vesicles, while the critical capillary number is approximately two times larger. Figure 2b shows that in this case the break-up is selective. Frames (i-ii) in Fig. 2b show the two populations of vesicles undergoing deformation in acoustic microstreaming flow (*p*_A_ = 17 kPa). The SOPC vesicles are fluorescently labelled, while the POPC vesicles are not. The fluorescence image in frame (ii) shows one SOPC vesicle undergoing strong deformation. The acoustic pressure is gradually increased in frames (iii) and (iv), up to *p*_A_ = 80 kPa. Frames (v–vi) are taken after the microstreaming flow has stopped (*p*_A_ = 0 kPa), and show that several large POPC vesicles (radius up to 23 *μ*m) have remained unaffected, while only very small SOPC vesicles are present. This observation is again consistent with the magnitudes of the capillary numbers. The graph in Fig. 2b shows that, for vesicles with radius *R* = 30 *μ*m, the capillary number for a characteristic stress *τ*_max _≈ 100 Pa is well below the threshold for break-up of POPC vesicles (open circle), while it matches the threshold for SOPC vesicles (solid circle). This selectivity is not present for radii below *R* = 15 *μ*m (open circles). In experiments on approximately 10 SOPC vesicles and 20 POPC vesicles, we observed probabilities of break-up of vesicles larger than 15 *μ*m in the same flow conditions of 85% and 20%, respectively. The observed selectivity is primarily due to the large difference in critical capillary numbers between SOPC and POPC vesicles. Selective rupture has been obtained by gradually increasing the stress, *τ*_max_, up to the value for which only one population reaches the threshold for rupture. If the stress is sufficiently large, vesicles from both population will break, even if the mechanical properties of the membrane are different. For the case of SOPC and POPC, we estimate from Eq. (1) that the stress above which all vesicles larger than *R* ≈ 15 *μ*m should break is *τ*_max_ ≈ 400 Pa. If the stress is further increased, rupture of smaller vesicles would also occur. The stress required for rupture of all vesicles larger than *R* ≈ 5 *μ*m is *τ*_max _≈ 1100 Pa. The maximum stress that can be achieved in our setup is *τ*_max_ ≈ 300 Pa.

### Selective rupture of vesicles with lipid:cholesterol membranes

Cholesterol modifies the ordering of the hydrocarbon tails in lipid bilayers and affects the mechanical properties of membranes, to an extent that depends on the degree of unsaturation of the lipids. For instance, the bending modulus, *κ*_b_, of mono-unsaturated lipids SOPC and POPC increases in the presence of cholesterol^10^,^25^, while that of double-unsaturated lipid DOPC is unaffected^25^. The stretching elasticity, *K*_*A*_, increases for both mono-unsaturated and double-unsaturated lipids, with a more pronounced effect on mono-unsaturated lipids^12^. The compositions used here, with a 1:1 molar ratio of lipids and cholesterol, give a seven-fold increase in *K*_*A*_ for SOPC, and a three-fold increase for DOPC (see Table 1). The critical capillary numbers are smaller for the lipid:cholesterol mixtures than for pure lipids, both in the case of SOPC and DOPC. This lower threshold for vesicle break-up is primarily due to the decrease in maximum area dilation, *α* (Table 1).

**Figure 3:**
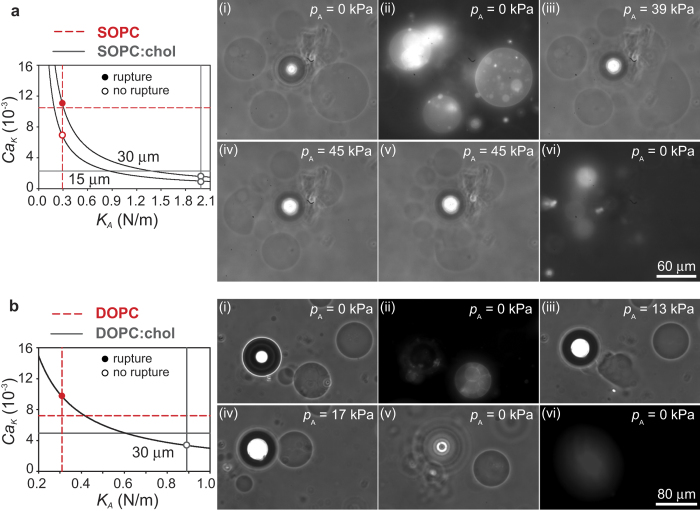
Selectivity of flow-induced vesicle break-up for lipid:cholesterol membranes. (**a**) SOPC (fluorescent) against SOPC:chol (1:1). Selectivity is observed in frames (i–vi), as only SOPC vesicles break up in the microstreaming flow. The graphs shows that 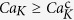 only for SOPC vesicles (dashed lines) but not for SOPC:chol vesicles (solid lines). (**b**) DOPC (fluorescent) against DOPC:chol (1:1). Break-up is selective, as shown in frames (i–vi) where, of two vesicles of identical size *R* ≈ 30 *μ*m, only the DOPC vesicle breaks. Selectivity is consistent with the difference in mechanical properties as shown in the graph. Dashed lines correspond to DOPC and solid lines to DOPC:chol. All images are in top view.

Figure 3a shows the selective break-up of SOPC vesicles in a suspension containing both SOPC and SOPC:chol (1:1) vesicles, exposed to the same microstreaming flow conditions. Frames (i-ii) show phase contrast and fluorescence images of the system before the microstreaming flow is applied. The SOPC vesicles are fluorescently labelled. The SOPC:chol vesicles are not fluorescent, and are only visible in (i). The ultrasound is applied and the acoustic pressure *p*_A_ is progressively increased to *p*_A_ = 39 kPa (iii) and up to *p*_A_ = 45 kPa (iv-v), resulting in the break-up of several large SOPC vesicles, and the release of smaller vesicles. SOPC vesicles smaller than *R* ≈ 15 *μ*m do not break up. The SOPC:chol vesicles are not visibly deformed by the microstreaming flow, as is clear in frames (iii-v), and they do not break. The graph in Fig. 3a shows that, for vesicles with radius *R* ≈ 30 *μ*m, the capillary number for a characteristic stress *τ*_max_ ≈ 100 Pa is above the threshold for break-up of SOPC vesicles (solid circle), and below the threshold for SOPC:chol vesicles (open circle). Neither population is expected to break for radii below *R* ≈ 15 *μ*m (open circles), consistent with experimental observations. We observed approximately 20 SOPC vesicles and 10 SOPC:chol vesicles, and found probabilities of break-up of vesicles larger than 15 *μ*m in the same flow conditions of 87% and 10%, respectively. This high level of selectivity is in keeping with the large difference is stretching elasticity between SOPC and SOPC:chol membranes (Table 1).

The increase in stretching elasticity upon addition of cholesterol is smaller for the case of DOPC. This difference is reflected in the probabilities of break-up for DOPC and DOPC:chol. We observe rupture of 87% of the DOPC vesicles, and 33% of the DOPC:chol vesicles, in the same flow conditions. The sample size is 15 vesicle for each population. Figure 3b shows an example with two vesicles of the same size, *R* ≈ 30 *μ*m. The fluorescent vesicle visible in frame (ii) is made of DOPC. This vesicle is clearly seen to break in frame (iii) for *p*_A_ = 13 kPa, while the DOPC:chol vesicle (not fluorescent) is exposed to an even stronger microstreaming flow for *p*_A_ = 17 kPa (iv), but does not break. Also in this case the scaling of the capillary number with *K*_*A*_ explains the selectivity in the rupture of vesicles with radius 30 *μ*m. The graph in Fig. 3b shows that DOPC vesicles are expected to break (solid circle), while DOPC:chol vesicles are not (open circle).

### Effect of variability in excess area on mechanical selectivity

**Figure 4:**
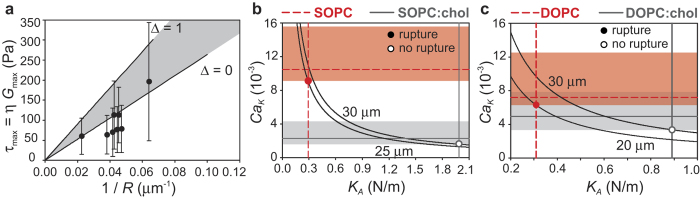
Effect of variability in excess area on mechanical selectivity of vesicle break-up. (**a**) Maximum viscous stress at break-up, *τ*_max_, as a function of the inverse of the vesicle radius, 1/*R*, for SOPC vesicles. The experimental points do not fall on a line of constant 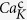 because the variability in excess area results in a range of values of 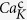 for the same vesicle population. The points fall in the shaded area indicating the range of 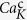 determined independently from excess area measurements. (**b**) Conditions for selective break-up of SOPC vesicles against SOPC:chol vesicles, taking into account the variability in 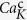. The range of vesicle radii in which selectivity is robust is *R* ≈ 25–30 *μ*m. (**c**) Conditions for selective break-up of DOPC vesicles against DOPC:chol vesicles, taking into account the overlap in thresholds for break-up due to the variability in 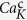. The range of vesicle radii in which selectivity is robust is *R* ≈ 20–30 *μ*m.

The selectivity of vesicle break-up with respect to the mechanical properties of the membrane, and the size threshold observed in the experiments of Figs 2 and 3, are qualitatively captured by the criterion based on the capillary number, 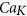, and the threshold value, 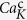
18. The values of 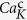 reported in Table 1 are calculated using the average value of the excess area for each vesicle population. However, in our measurements of excess area (see *Methods*) we find a distribution of values of Δ, which results in a certain degree of variability of the critical capillary number for vesicles of the same population. We studied how the variability in excess area affects the rupture conditions for the case of SOPC vesicles. Figure 4a reports the results of break-up experiments in which the acoustic pressure *p*_A_ was sufficiently small that the bubble oscillations remained spherical. To obtain sufficiently large viscous stresses to promote vesicle break-up even for small-amplitude oscillations, we increased the viscosity of the solution (*η* = 30 mPa s or *η* = 100 mPa s) using water/glycerol mixtures (see *Methods*). In this regime we are able to estimate the maximum strain rate, *G*_max_, from the measured amplitudes of radial and translational oscillations, *ε*′ and 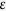, using Eq. (1). The break-up condition 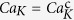 is recast as 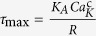, with *τ*_max_ the maximum viscous stress at rupture, *τ*_max_ = *ηG*_max_. Each experimental data point corresponds to a vesicle break-up event, characterized by the vesicle radius *R* and the value of *G*_max_ obtained from Eq. (1). The error bars account for the typical inaccuracy introduced by using Equation (1) (see Fig. 1f). The values of *τ*_max_ for the rupture of SOPC vesicles range from 50 to 200 Pa, motivating the assumption made in the analysis of Figs 2 and 3 of a characteristic stress for selective break-up of the order of 100 Pa. For a given value of 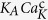, a linear relationship between the maximum stress and the inverse of the radius is expected. The values of excess area for SOPC vesicles, as obtained from capillary flow experiments (see *Methods*), fall in the interval Δ = 0–1, corresponding to a range of critical capillary numbers 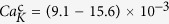. The shaded area in Fig. 4a represents this range, with the solid lines marking the limiting values of 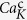 for Δ = 0 and Δ = 1. The data points obtained from break-up events do indeed not fall on a line of constant 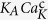, but they are scattered and fall, to within the experimental error, in the range of critical capillary numbers expected from the excess area measurements. Many of the vesicles are found to exhibit values of excess area at the lower end of the range Δ = 0–1, consistent with the excess area distributions found independently in the capillary flow experiments (see *Methods*).

The variability in critical capillary number, resulting from the variability in excess area within a vesicle population, can affect mechanical selectivity. Figure 4b shows how the variability in 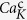 influences selectivity for the case of SOPC and SOPC:chol, for which good selectivity is observed (87% and 10% probability of rupture for vesicles larger than 15 *μ*m in the same flow conditions). The respective ranges of 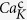, represented by shaded horizontal areas, do not overlap. A range of vesicle sizes can be identified for which robust selectivity should be observed: the solid line for *R* = 25 *μ*m is the lower limit of this size range, and it corresponds to the minimum threshold for rupture of SOPC vesicles, which is below the threshold for rupture of SOPC:chol. The upper limit is *R* = 30 *μ*m, corresponding to the minimum threshold for break-up of SOPC:chol vesicles, and within the range of 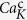 for rupture of SOPC vesicles. Figure 4c shows that for the case of DOPC and DOPC:chol, for which selectivity is slightly less effective (87% and 33% probability of rupture for vesicles larger than 15 *μ*m in the same flow conditions), the respective ranges of 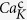 have some overlap. Nevertheless, also in this scenario a range of vesicle sizes can be identified for which complete selectivity should be observed. Below *R* = 20 *μ*m neither vesicle population breaks, while *R* = 30 *μ*m is the maximum size for which only DOPC vesicles break. The overlap between the respective ranges of 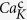 would prevent selectivity only if the two values of the stretching elasticity were sufficiently close.

In summary, we have identified conditions in which the selectivity of flow-induced vesicle break-up based on the membrane stretching elasticity is robust. We have investigated the effect of membrane stretching elasticity, vesicle radius, and excess area on break-up conditions. The observed size threshold for break-up is in keeping with the dependence of the capillary number on the inverse of the radius. The variability in excess area results in a variability of the threshold for vesicle break-up within the same vesicle population. It is possible to identify conditions for which selectivity is robust despite the variability in excess area. In particular, a range of vesicles sizes exists for which the capillary number for one population exceeds the threshold for break-up, while the capillary number for the other population does not. This condition can be met either if the critical capillary numbers differ significantly, or if the stretching elasticities differ significantly. We have tested these break-up conditions both on single-component vesicles made of SOPC, DOPC, or POPC, and on vesicles containing cholesterol, to highlight the fact that the effect is purely mechanical and independent of the particular composition. These observations open the way to new bioprocessing and drug delivery methods based on the difference in membrane mechanical properties between soft deformable objects such as vesicles, capsules and cells, for instance between healthy cells and cancer cells^29^.

## Methods

### Preparation of giant unilamellar lipid vesicles

Giant unilamellar lipid vesicles were made using standard electroformation protocols^32^. The lipids used were 1-stearoyl-2-oleoyl-*sn*-glycero-3-phosphocholine (SOPC), 1,2-dioleoyl-*sn*-glycero-3-phosphocholine (DOPC), and 1-palmitoyl-2-oleoyl-*sn*-glycero-3-phosphocholine (POPC). We made single-component vesicles of pure SOPC, DOPC or POPC. We made two-component vesicles using lipid:cholesterol mixtures: SOPC:cholesterol in a 1:1 molar ratio; and DOPC:cholesterol in a 1:1 molar ratio. Vesicles were fluorescently labelled by adding either rhodamine-tagged lipid 1,2-dioleoyl-sn-glycero-3-phosphoethanolamine-N-(lissamine rhodamine B sulfonyl) (ammonium salt), or NBD-tagged lipid 1,2-dioleoyl-sn-glycero-3phosphoethanolamine-N-(7-nitro-2-1,3-benzoxadiazol-4-yl) (ammonium salt), in a 1:100 molar ratio. SOPC, DOPC, POPC and fluorescently tagged lipids were purchased from Avanti Polar Lipids and used without further purification. Cholesterol was purchased from Sigma-Aldrich and used without further purification. Lipid solutions (1 mg/ml in chloroform) were first dried under vacuum (1 h), and subsequently hydrated in an aqueous sucrose solution in a custom-made electroformation chamber composed of two transparent electrodes (ITO glass, Sigma-Aldrich) and a PDMS spacer (1 mm thick). The AC voltage amplitude and frequency, and the duration of the hydration step, were optimised depending on the solution to encapsulate. For the preparation of vesicles in pure water the AC voltage was set to 1.1 V at 10 Hz for 3 hours, followed by 4.4 V at 4 Hz for 45 minutes. We also used water/glycerol mixtures with a 0.45/0.55 mass ratio to obtain a viscosity *η* = 30 mPa s, and a 0.33/0.67 mass ratio to obtain a viscosity *η* = 100 mPa s. For the preparation of vesicles in water/glycerol mixtures, a voltage of 1.1 V at 7 Hz was set overnight^33^. In all cases the electroformation solution contained 0.11 M sucrose, and after electroformation the vesicles were transferred to solutions containing 0.12 M glucose. The viscosity ratio between the inner and outer solution was equal to 1 in all the experiments. Sucrose and glucose were purchased from Sigma-Aldrich and used without further purification. The vesicles produced with this method ranged from a few micrometers up to 50 *μ*m in radius.

### Acoustic microstreaming flow setup

The setup for vesicle deformation and break-up is similar to the one used in Ref. 17. A piezoelectric transducer (Physik Instrumente) was glued to the wall of a quartz cuvette. The transducer was driven by the signal produced by a waveform generator (Agilent) and amplified by a linear radio-frequency power amplifier (T&C Power Conversion). The frequency of the ultrasound wave, *f*, can be selected in the range 50–200 kHz. The cuvette was filled with the suspension of vesicles. Air microbubbles of 10–100 *μ*m in radius were formed with a syringe and adhered to the cuvette walls. The pressure fluctuations created by the ultrasound wave, *p*(*t*) = *p*_A_ cos(*ωt*), with *p*_A_ the acoustic pressure amplitude and *ω* = 2*πf* the angular frequency, cause the microbubbles to periodically compress and expand. The relative amplitude of radial oscillations, *ε*′, depends on the driving frequency, being a maximum at the resonance frequency of the bubble, and on the acoustic pressure amplitude^34^. The frequency of the ultrasound wave was selected to match the resonance frequency of the microbubble. For small-amplitude oscillations, the shape of the bubbles remains approximately spherical. The radial oscillations are accompanied by translational oscillations of the center of mass perpendicular to the solid wall where the bubble is attached^30^, with relative amplitude *ε*. The radial and translational oscillations result in a non-linear secondary flow known as acoustic microstreaming^30^. We observed the oscillatory bubble dynamics using a high-speed camera (Photron FASTCAM SA5) at 300,000 frames per second, and the steady microstreaming flow using a high-resolution camera at 50 frames per second. The acoustic pressure inside the cuvette was measured using a PVDF hydrophone (RP Acoustics).

### Measurements of excess area distributions

We measured the excess area Δ by observing vesicle deformation in a capillary flow. The capillaries used were cylindrical silica microcapillaries (Polymicro Technologies) with an internal diameter of 100 *μ*m, resulting in a degree of confinement, given by the ratio of vesicle diameter to capillary diameter, ranging from 0.14 to 0.36. Immersion oil (Sigma Aldrich, refractive index 1.51) was used to match the refractive index of silica. Image analysis was used to measure the deformation of vesicles flowing in the midsagittal plane. Because of the refractive index mismatch between the glucose solution (*n*_s_ ≈ 1.335) and silica (*n*_g_ ≈ 1.5), refraction of light at the curved solid/liquid interface introduces distortions in the observed vesicle shapes. The effect of the curvature of microcapillaries with radius *r* has been predicted from Snell’s law^35^. The real distance *d*_a_ of an object in the midsagittal plane from the capillary wall can be obtained from the measured distance *d*_m_ using the equation *d*_a_ = *d*_m_ − *δx*, where


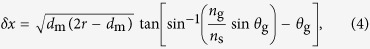


and


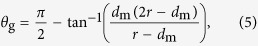


with *θ*_g_ the angle of incidence. In this way the apparent decrease of length along the radial direction is corrected and the real deformation of vesicles is measured.

**Figure 5:**
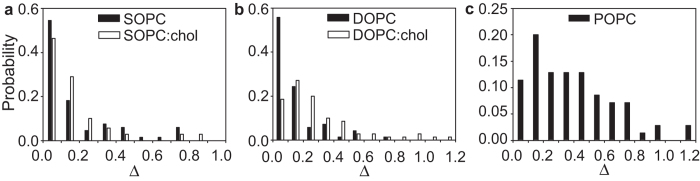
Excess area distributions of the five vesicle populations as determined from capillary flow experiments. (**a**) Distributions of excess area for SOPC and SOPC:chol vesicles. (**b**) Distributions of excess area for DOPC and DOPC:chol vesicles. (**c**) Distributions of excess area for POPC vesicles. Size of sample was *N* ≈ 70 for all populations.

A syringe pump (Harvard Apparatus) was used to push the vesicle solution into the microcapillary with a flow rate ranging from 10 to 1000 nL/s. The flow velocity *U* was set to completely flatten the vesicle membranes without stretching them (see Fig. 1g). To fulfill this condition, the capillary number describing the resistance to bending in a Poiseuille flow needs to satisfy^36^ *Ca*_b_ = *ηUR*^2^/*κ*_b_ > 1, while the capillary number based on the stretching elasticity, *Ca*_*K*_ = *ηU*/*K*_*A*_, needs to satisfy^17^ 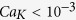. For our measurements we used flow velocities such that 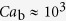 and 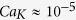. Vesicles that were not flowing on the axis of the capillary deformed assuming an ellipsoidal shape. We measured the two semi-axes and, assuming the three-dimensional shape to be a prolate ellipsoid, we computed the volume and surface area of the vesicles. We then computed the surface area of a sphere with the same volume to obtain the excess area Δ. We analyzed approximately 70 vesicles for each population and obtained the five excess area distributions shown in Fig. 5. The mean values of excess area are Δ_SOPC_ = (0.17 ± 0.21), Δ_SOPC:chol_ = (0.17 ± 0.24), Δ_DOPC_ = (0.13 ± 0.16), Δ_DOPC:chol_ = (0.31 ± 0.31), Δ_POPC_ = (0.39 ± 0.27).

## Additional Information

**How to cite this article**: Pommella, A. *et al.* Selective flow-induced vesicle rupture to sort by membrane mechanical properties. *Sci. Rep.* **5**, 13163; doi: 10.1038/srep13163 (2015).

## Figures and Tables

**Table 1:** Vesicle properties for the five membrane compositions used.

	**SOPC**	**DOPC**	**POPC**	**SOPC:chol (1:1)**	**DOPC:chol (1:1)**
*κ*_b_ (×10^−19^) [J]	0.9	1.08	1.58	2.46	1.08
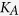 [mN/m]	290	310	198	1985	890
*α* = *σ*_c_/*K*_*A*_	0.041	0.032	0.063	0.013	0.021
Δ	0.17	0.13	0.39	0.17	0.31
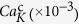	10.49	7.22	21.12	2.31	4.93

The values of the bending modulus, *κ*_b_, the stretching elasticity, *K*_*A*_, and the maximum area dilation *α* = *σ*_c_/*K*_*A*_, where *σ*_c_ is the lysis tension, are taken from the literature: *κ*_b_ of SOPC and SOPC:chol (1:1) from Ref. 10; *K*_*A*_ and *σ*_c_ of SOPC and SOPC:chol (1:1) from Ref. 12; *κ*_b_ of DOPC and DOPC:chol (1:1) from Ref. 25; *K*_*A*_ and *σ*_c_ of DOPC and DOPC:chol (1:1) from Ref. 12; *κ*_b_ of POPC from Ref. 24; *K*_*A*_ and *σ*_c_ of POPC from Ref. 26. The excess area, Δ, was measured in capillary flow experiments (see *Methods*). The values reported are the mean for each population. The critical capillary number, 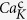, is calculated from *α* and Δ.
